# Supplementary material for: Complexome profiling of the Chlamydomonas psb28 mutant reveals TEF5 as an early PSII assembly factor
Source: Plant Cell. 2025 Mar 17;37(6):koaf055. doi: 10.1093/plcell/koaf055 (PMC12164590; doi:10.1093/plcell/koaf055)
Supplement: koaf055_Supplementary_Data [file koaf055_supplementary_data.zip › psb28_interactive.zip!/plots/intensity/qualityPlots/peptides/_Cre16.g651923.t1.2.html]

### Cre16.g651923.t1.2

Trivial name:   
SeqCoverage [median]: 39.10  
Mass: 66.91 kDa  
  
 

| Sequence | MissedCleavage | Mass [Da] | wtIntensity | muIntensity | Score |
| --- | --- | --- | --- | --- | --- |
| AFGALLR | 0 | 746.44 | 2255.37 | 5172.33 | 103.41 |
| ANVKEILLSPQPDGSQR | 1 | 1850.98 | 623.70 | 725.92 | 53.56 |
| DKLPESEQLFR | 1 | 1360.70 | 3474.62 | 10377.35 | 120.55 |
| EDGTYGPIPSR | 0 | 1190.56 | 1508.99 | 1830.31 | 82.75 |
| EILLSPQPDGSQR | 0 | 1438.74 | 7249.45 | 12323.15 | 143.96 |
| FLNREDGTYGPIPSR | 1 | 1720.85 | 4636.53 | 12397.08 | 78.93 |
| GLEVAVWR | 0 | 928.51 | 3650.81 | 6468.77 | 108.24 |
| HFGGINYPVGGVGR | 0 | 1428.73 | 1400.36 | 6488.21 | 59.15 |
| IETVPDPTQVVYHLPK | 0 | 1834.98 | 907.15 | 6359.04 | 91.44 |
| IFDSLNVLDLK | 0 | 1275.71 | 3290.99 | 0.00 | 123.63 |
| IGEELAAGIEEYGGK | 0 | 1534.75 | 9957.70 | 20840.54 | 167.84 |
| KIETVPDPTQVVYHLPK | 1 | 1963.08 | 3176.76 | 13458.21 | 88.99 |
| KYEEFIDELAAK | 1 | 1454.73 | 5132.25 | 15593.20 | 108.72 |
| KYEEFIDELAAKFPHER | 2 | 2121.05 | 568.14 | 0.00 | 24.79 |
| LPESEQLFR | 0 | 1117.58 | 0.00 | 1312.00 | 69.60 |
| RLDAILPGLSSNITFR | 1 | 1771.99 | 769.71 | 0.00 | 55.55 |
| SLEEPR | 0 | 729.37 | 1111.20 | 2598.47 | 102.98 |
| SPSFFSIHMGVK | 0 | 1335.66 | 3288.90 | 8962.91 | 76.65 |
| TVISNATR | 0 | 860.47 | 3615.53 | 15661.59 | 111.95 |
| VINTEAPTDVEYDAVIVGGGMGGLATAAR | 0 | 2846.41 | 2460.97 | 5189.24 | 70.47 |
| VLTDLGREPAWPVIDK | 1 | 1807.98 | 2101.68 | 5373.54 | 65.70 |
| YLLGEFAK | 0 | 939.51 | 1623.49 | 10474.85 | 79.12 |
| YLLPGGSAAHFK | 0 | 1259.67 | 1933.31 | 13592.60 | 82.17 |
| YLLPGGSAAHFKR | 1 | 1415.77 | 0.00 | 848.56 | 38.63 |
|  |  |  | 64737.6 | 176047.9 |  |
